# Characterization of Phase-Based Methods Used for Transmission Field Uniformity Mapping: A Magnetic Resonance Study at 3.0 T and 7.0 T

**DOI:** 10.1371/journal.pone.0057982

**Published:** 2013-03-05

**Authors:** Flavio Carinci, Davide Santoro, Federico von Samson-Himmelstjerna, Tomasz Dawid Lindel, Matthias Alexander Dieringer, Thoralf Niendorf

**Affiliations:** 1 Berlin Ultrahigh Field Facility (BUFF), Max Delbrück Center for Molecular Medicine (MDC), Berlin, Germany; 2 Research Center Magnetic Resonance Bavaria (MRB), Würzburg, Germany; 3 Center for Stroke Research Berlin (CSB), Charitè Universitätsmedizin, Berlin, Germany; 4 Institute for Medical Image Computing, Fraunhofer MEVIS, Bremen, Germany; 5 Department of Medical Metrology, Physikalisch Technische Bundesanstalt (PTB), Berlin, Germany; 6 Experimental and Clinical Research Center (ECRC), a joint cooperation between the Charité Medical Faculty and the Max Delbrück Center for Molecular Medicine (MDC), Berlin, Germany; University of Minnesota, United States of America

## Abstract

Knowledge of the transmission field (*B_1_* ^+^) of radio-frequency coils is crucial for high field (*B_0_* = 3.0 T) and ultrahigh field (*B_0_*≥7.0 T) magnetic resonance applications to overcome constraints dictated by electrodynamics in the short wavelength regime with the ultimate goal to improve the image quality. For this purpose *B_1_* ^+^ mapping methods are used, which are commonly magnitude-based. In this study an analysis of five phase-based methods for three-dimensional mapping of the *B_1_* ^+^ field is presented. The five methods are implemented in a 3D gradient-echo technique. Each method makes use of different RF-pulses (composite or off-resonance pulses) to encode the effective intensity of the *B_1_* ^+^ field into the phase of the magnetization. The different RF-pulses result in different trajectories of the magnetization, different use of the transverse magnetization and different sensitivities to *B_1_* ^+^ inhomogeneities and frequency offsets, as demonstrated by numerical simulations. The characterization of the five methods also includes phantom experiments and *in vivo* studies of the human brain at 3.0 T and at 7.0 T. It is shown how the characteristics of each method affect the quality of the *B_1_* ^+^ maps. Implications for *in vivo B_1_* ^+^ mapping at 3.0 T and 7.0 T are discussed.

## Introduction

Non-uniformities of the transmission radio-frequency (RF) field (*B_1_* ^+^) constitute an adverse factor for high field (*B_0_* = 3.0 T) and ultrahigh field (*B_0_*≥7.0 T) magnetic resonance (MR), which may render diagnostics challenging. This practical impediment is pronounced when imaging techniques sensitive to the excitation flip angle (FA) are applied. The knowledge of the *B_1_* ^+^ field distribution is essential to correct for *B_1_* ^+^ non-uniformities of single channel or multi-channel transmit (TX) RF-coils. To trim or shim the *B_1_* ^+^ field, multiple channel transmission has been pioneered [1]–[3]. For this purpose, multi transmit arrays are used, which require *B_1_^+^* mapping routines to calibrate each individual RF coil element. This procedure can be time consuming when using TX arrays comprising many transmit elements. Consequently accurate and fast *B_1_^+^* distribution mapping is the key for ultrahigh field clinical applications.

*B_1_^+^* mapping approaches commonly used are mainly magnitude-based and are generally confined to the ratios or the fit of signal intensity images [4]–[11]. For this purpose sets of images are acquired using either two flip angles [4]–[6], identical flip angles but different repetition times (TR) [7], variable flip angles [8], [9] or also signals from spin-echoes and stimulated-echoes [10], as well as signals from gradient-echoes and stimulated-echoes [11]. For most of these magnitude-based approaches the quantitative *B_1_^+^* evaluation may be influenced by saturation effects given by *T_1_* relaxation. This problem can be overcome with the use of long repetition times (TR), which, however, would result in prolonged acquisition times. Alternatively, phase-based methods have been proposed as they are insensitive to *T_1_* relaxation. They were also found to be more accurate than magnitude-based methods, especially at low flip angle regimes [12].

Realizing the advantages of phase-based methods for *B_1_^+^* mapping, this work characterizes five of these methods: **A)** an optimized version for high field proton MRI [13] of the low flip angle method proposed by Mugler [14], [15] here named “*Optimized low-flip-angle method*”, **B)** the phase sensitive method of Morrell [16] here named “*Phase-sensitive method*”, **C)** the phase-based method of Santoro [17], [18], applied to high field proton MRI [19] here named “*ФFA-CUP method*”, **D)** the Bloch-Siegert shift method of Sacolick [20]–[22] here named “*Bloch-Siegert method*” and **E)** the orthogonal pulses method proposed by Chang [23] here named “*Orthogonal-pulses method*”. These phase-based methods share in common the use of a composite or off-resonance RF-pulse to encode the spatial *B_1_^+^* magnitude information into the phase of the magnetization vector (**M**). Each method uses a different scheme of the RF-phases, generating a different evolution of **M**. The sensitivity to *B_1_^+^* variations and frequency offsets is examined using numerical simulations of the Bloch equations. Phantom experiments and human brain imaging studies are conducted at 3.0 T and 7.0 T to scrutinize each method. This includes the assessment of repetition times achievable, according to specific absorption rate (SAR) levels, as well as the susceptibility to off-resonance effects. For a balanced comparison, all methods are used in conjunction with the same reading module.

## Materials and Methods

### Theory

**Figure 1:**
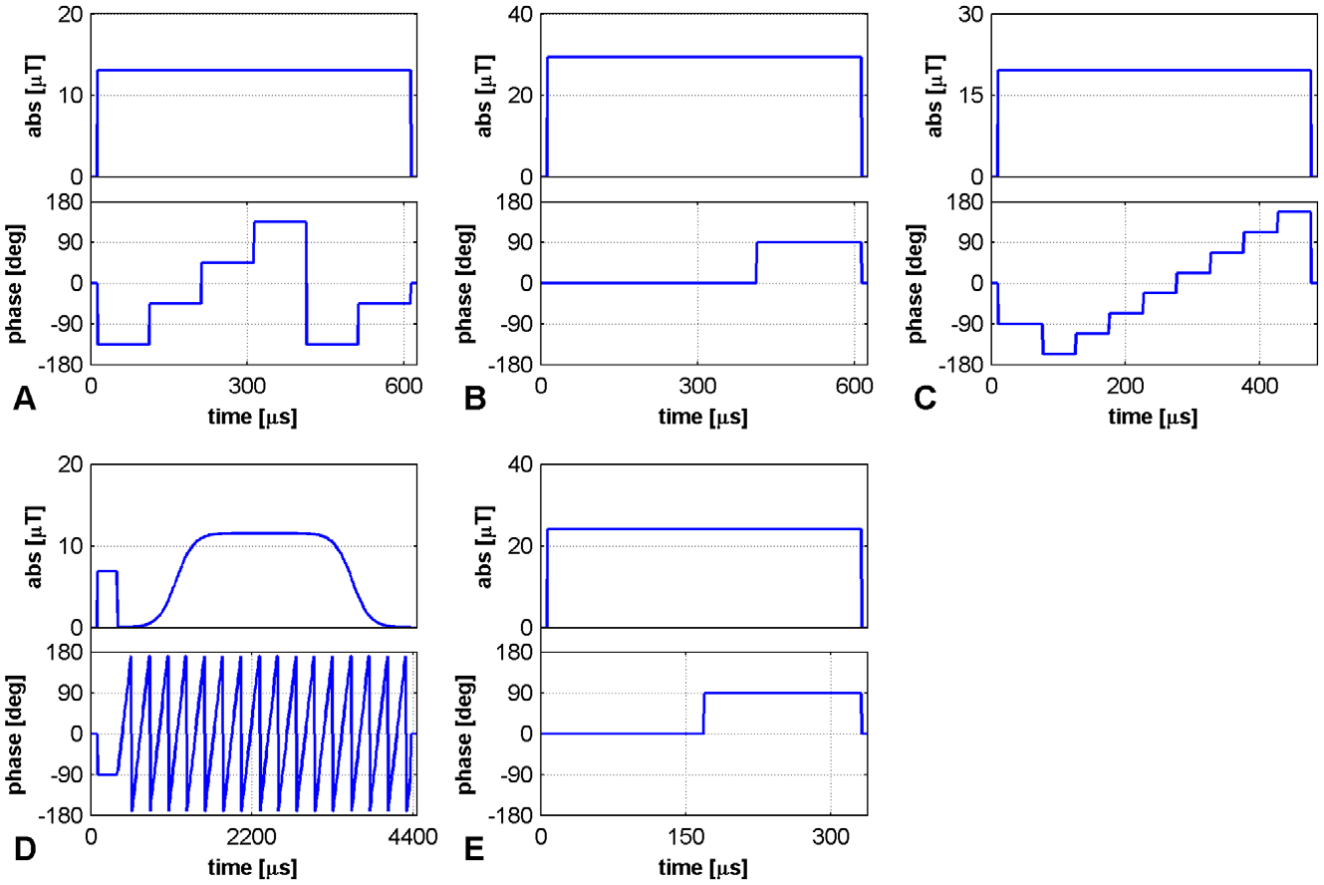
RF-pulse envelopes for phase-based *B_1_^+^* mapping. Diagrams of the composite pulses used for all methods: **A –** *Optimized low-flip-angle method* **, B –** *Phase-sensitive method* ***,*** **C –** *ΦFA-CUP method* **, D –** *Bloch-Siegert method*, **E –** *Orthogonal-pulses method*. Pulse timing, intensity of the *B_1_^+^* field (in µT) and RF-phases (in degrees) are sketched. The parameters used are: **A)** α = 20° and duration of 600 µs; **B)** α = 90° and duration of 600 µs; **C)** *p0* = 20°, α = 15° and duration of 400 µs (plus the duration of the *p0* pulse); **D)** *p0* = 20°, α = 425° and duration of 4000 µs (plus the duration of the *p0* pulse); **E)** α = 60° and duration of 325 µs.

**Figure 2:**
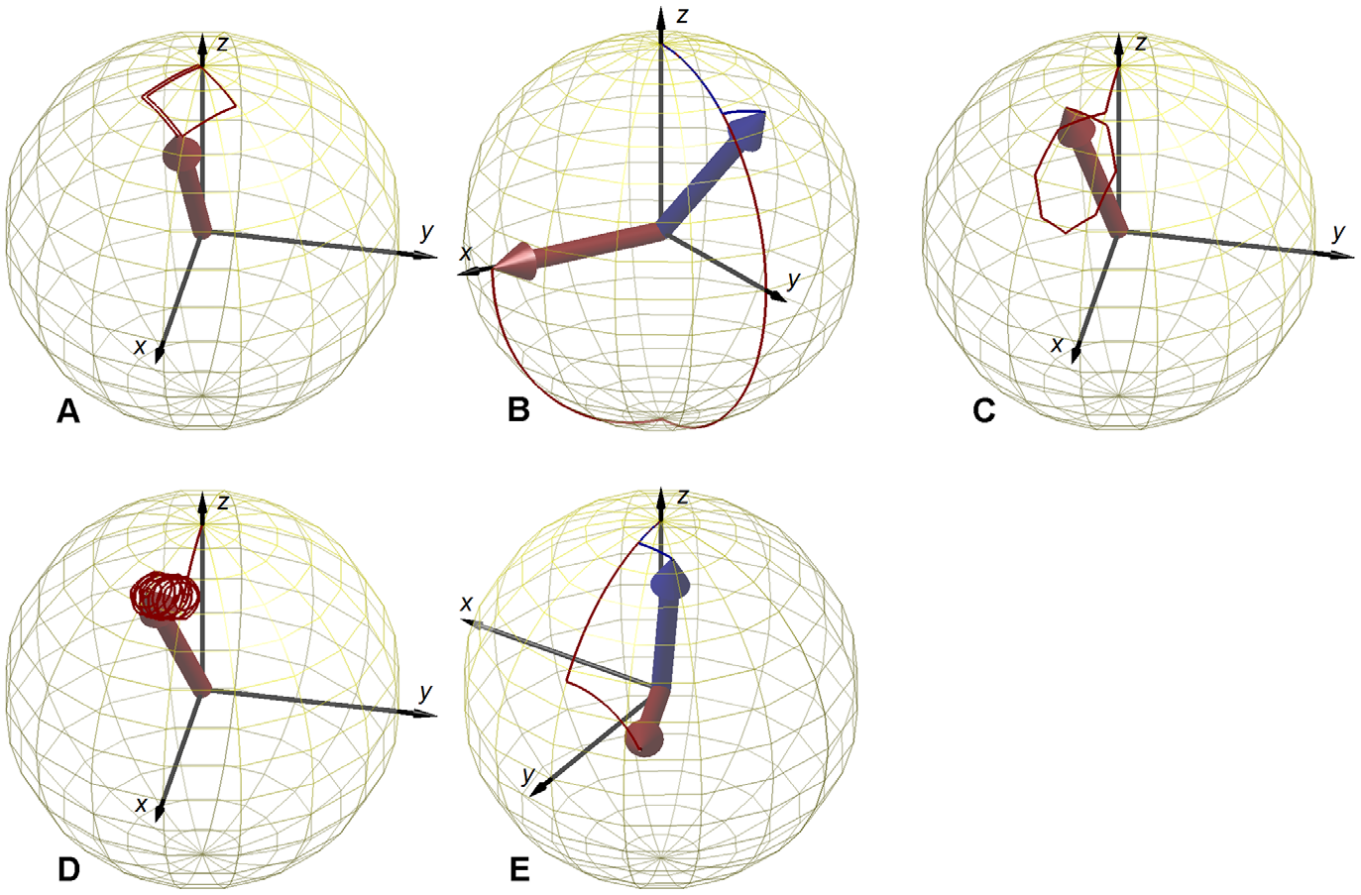
Trajectories of the magnetization during RF-excitation. Evolution of **M** in a unitary sphere during the RF-pulses of Fig.1 (red lines), under ideal conditions (Δ*B_0_* = 0) for all methods: **A –** *Optimized low-flip-angle method* **, B –** *Phase-sensitive method* ***,*** **C –** *ΦFA-CUP method* **, D –** *Bloch-Siegert method*, **E –** *Orthogonal-pulses method*. **A)** a squared trajectory is traversed for one and a half turns; **B)** for small flip angles a rectangular trajectory is traversed for half turn (blue line: α = 18°), the flip angle originally proposed moves **M** into the transverse plane (red line: α = 90°); **C)** an intial pulse moves **M** far from the origin, then an octagonal loop is traversed for one turn only; **D)** an initial excitation is followed by an off-resonance pulse, which is equivalent to traversing a circular trajectory for several turns; **E)** for small flip angles a square trajectory is traversed for half turn (blue line: α = 12°), the flip angle originally proposed moves **M** close to the transverse plane (red line: α = 60°).

The *B_1_^+^* mapping methods analyzed in this work make use of a *complex* RF-pulse envelope (a rectangular composite pulse or an off-resonance Fermi pulse) for excitation, with separately controlled amplitude and phase (Fig. 1). Each pulse achieves a different trajectory of the magnetization **M**, depending on the combination of amplitude and phase of the RF-pulse. The trajectories of **M** for the five pulses are depicted in Fig. 2 for the ideal case where Δ*B_0_* = 0. The presence of *B_0_* inhomogeneities, or other sources of frequency offsets, results in deviations from the ideal trajectory.

All the five trajectories can be represented by a different polygon lying on the surface of a unitary sphere. Each of them is characterized by a different number of sides and it is traversed a different number of times: **A** – ***Optimized low-flip-angle method*** **)** a squared trajectory which is traversed for one and a half turns [13]–[15], **B** – ***Phase-sensitive method*** **)** a rectangular trajectory which is traversed for a half turn [16], **C** – ***ФFA-CUP method*** **)** an off-origin loop trajectory which is traversed for a single turn [17]–[19], **D** – ***Bloch-Siegert method*** **)** an initial excitation followed by an off-resonance pulse (which is equivalent to traversing a small circular trajectory for several turns) [20]–[22] and **E** – ***Orthogonal-pulses method*** **)** a square trajectory which is traversed for a half turn [23].

**Figure 3:**
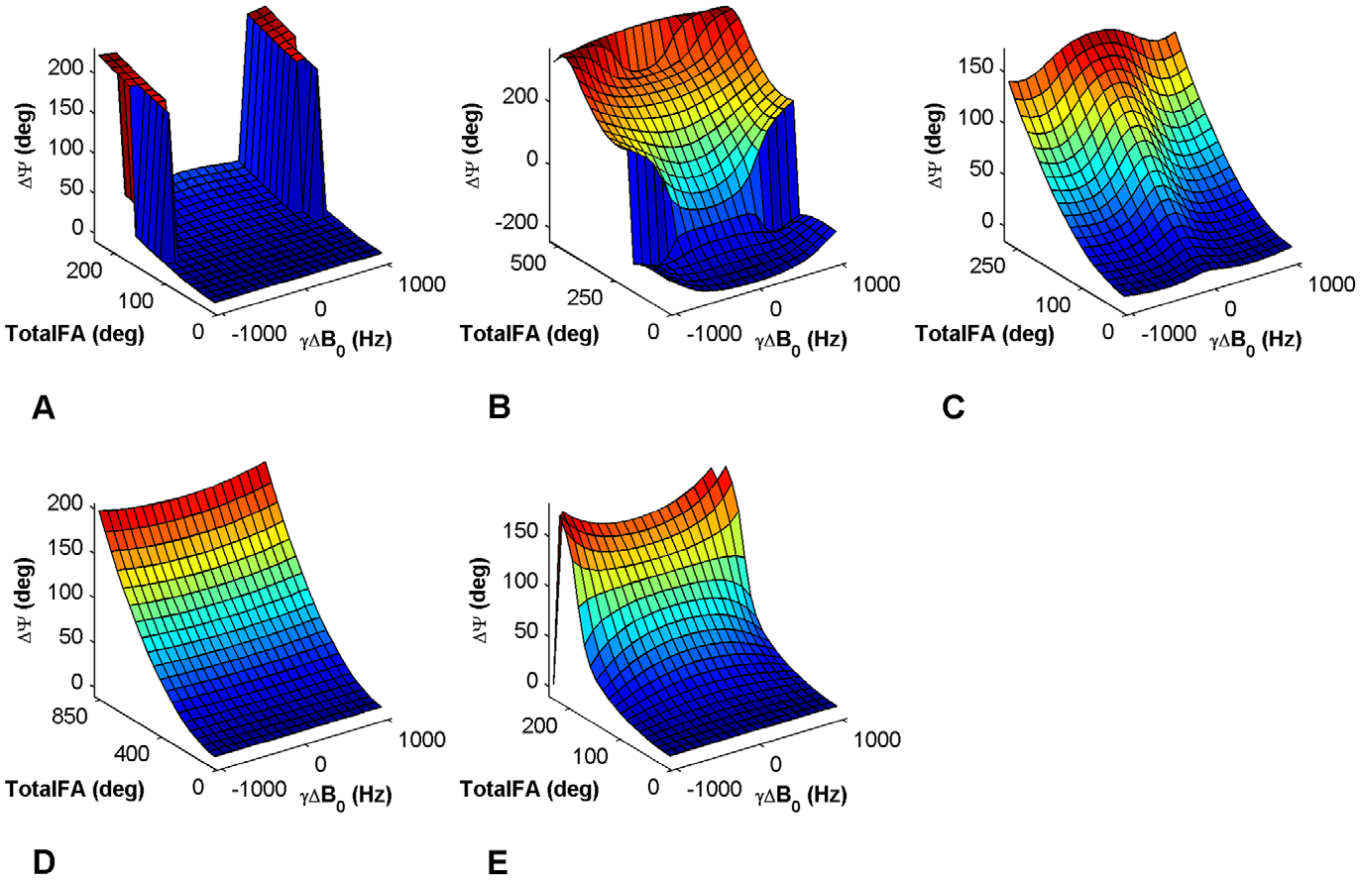
Curves of sensitivity to *B_1_^+^* and *B_0_* inhomogeneities. Phase accrual curves of the five methods plotted versus the *TotalFA* of the RF-pulses and a frequency offset distribution −1 kHz ≤ Δ*B_0_*≤1 kHz. The frequency offset range is the same for all methods, while the *TotalFA* ranges differ, as well as the phase accrual Ψ. These curves were derived from simulations (using the same parameters as in Fig. 1) and used for the 2D interpolation to obtain the *B_1_^+^* maps. Methods: **A –** *Optimized low-flip-angle method* **, B –** *Phase-sensitive method* ***,*** **C –** *ΦFA-CUP method* **, D –** *Bloch-Siegert method*, **E –** *Orthogonal-pulses method*.

At the end of each RF-pulse, the local magnetization presents a phase accrual depending on the local *B_1_^+^* intensity and frequency offset experienced, as shown in the curves of Fig. 3. The theoretical description of this effect has been already reported in [16], [18], [20], [23], and is briefly resumed here.

For methods **A**, **B**, **C** and **E** the RF-pulse can be divided into sub-pulses of flip-angle α and RF-phase Ф, denoted with α_Ф_. Each sub-pulse α_Ф_ represents a rotation about a different axis, due to their different RF-phase. The magnetization accumulates a phase shift which is proportional to the local *B_1_^+^* field intensity because of the non-commutativity of rotations about different axes.

Method **D,** after the initial excitation, uses an off-resonance RF-pulse. In this case the RF-phase varies linearly within the pulse and the magnetization accumulates a phase shift proportional to the local *B_1_^+^* field intensity, due to the well-known Bloch-Siegert shift effect [24].

To cancel phase contributions due to sources other than *B_1_^+^*, such as the receive coil sensitivity (*B_1_^−^*), the acquisition of two phase images, obtained with opposite senses of rotation of the magnetization (opposite RF-phase schemes), is required for all methods. The subtraction of the images preserves the *B_1_^+^* information, while removing all other time-independent phase contributions.

#### A – Optimized low-flip-angle method

Excitation is performed by the application of the non-selective composite pulse: [α_−135_ α_−45_ α_45_ α_135_ α_−135_ α_−45_] (Fig. 1A). The pulse moves the magnetization vector about a square, of side length α, through 1.5 turns (Fig. 2A) [13]. A second image must be acquired using a corresponding pulse that moves the magnetization in the opposite sense of rotation: [α_−45_α_−135_α_135_α_45_α_−45_α_−135_].

#### B – Phase-sensitive method

Excitation is performed by the application of the non-selective composite pulse: [2α_0_ α_90_] (Fig. 1B). For a small flip angle α the pulse moves the magnetization vector along a rectangular trajectory, with one side of length 2α and the other of length α, through 0.5 turns (Fig. 2B - blue line). The method is originally proposed using a nominal flip angle α = 90° (which performs the trajectory in Fig. 2B - red line) [16]. A second image must be acquired with the first sub-pulse reversed in sign: [2α_180_ α_90_].

#### C – ФFA-CUP method

Excitation is performed by the application of the non-selective composite pulse: [*p0*_−90_ α_−157.5_ α_−112.5_ α_−67.5_ α_−22.5_ α_+22.5_ α_+67.5_ α_+112.5_ α_+157.5_] (Fig. 1C). The magnetization vector is moved away from the origin by the first sub-pulse, named *p0*. The phase accrual is achieved by traversing for 1.0 turn an octagonal trajectory of side α shifted from the origin. The use of the first pulse *p0* separates the excitation from the phase accrual in order to optimize the sensitivity to *B_1_^+^* variations [17], [19]. A second image must be acquired with the composite pulse: [*p0*_+90_ α_+157.5_ α_+112.5_ α_+67.5_ α_+22.5_ α_−22.5_ α_−67.5_ α_−112.5_ α_−157.5_].

#### D – Bloch-Siegert method

This method makes use of an off-resonance pulse of frequency shift Δω_RF_ applied immediately after an excitation: [*p0*_−90_ α_−90,Δω_] (Fig. 1D). The off-resonance pulse moves the magnetization about a circular trajectory traversed several times (Fig. 2D). The number of loops is given by the duration of the pulse multiplied by the off-resonance frequency. The off-resonance pulse can be seen as a pulse in which the RF phase Ф is continuously varied during its duration τ, according to Ф = Δω_RF·_τ. A second image must be acquired using the opposite frequency shift:

-Δω_RF_. The method is originally proposed using an off-resonance Fermi pulse of frequency shift Δω_RF_ = 4 kHz and duration of 8 ms [20]. However it has been widely shown [21], [22] that different values of the pulse duration and frequency shift, as well as different pulse shapes, can be used to optimize this method. Here we used a Fermi pulse of frequency shift Δω_RF_ = 4 kHz and duration of 4 ms.

#### E – Orthogonal-pulses method

Excitation is performed by the application of the non-selective composite pulse: [α_0_ α_90_] (Fig. 1E). For a small flip angle α the pulse moves the magnetization vector along a square trajectory, with side of length α, through 0.5 turns (Fig. 2E – blue line). The method is originally proposed using a nominal flip angle α = 60° (which performs the trajectory in Fig. 2E - red line) [23]. A second image must be acquired with the phases of the two sub-pulses swapped: [α_90_ α_0_].

### Numerical Simulations

MATLAB (MathWorks Inc, Natick, USA) software was used to calculate the dynamics of the magnetization during the excitation pulses, by means of numerical simulations of the Bloch equations. A range of values of the frequency offset (−1 kHz ≤ Δ*B_0_*≤1 kHz, with an increment of 50 Hz) and of the *B_1_^+^* intensity (rescaling the flip angle from 0 to 2 times the nominal value, with an increment of 0.05) was used. The sensitivity of the different methods to the local variations of the *B_1_^+^* field and of the frequency offset is expressed by the variable Ψ (Fig. 3), which is defined as the subtraction of the phase accruals obtained from the two complementary scans required by each method. The intensity of the *B_1_^+^* is expressed in terms of the total flip angle (*TotalFA*) used by each pulse, which results from the total duration and amplitude of the RF applied, regardless of its RF-phase scheme.

At high field strengths the *TotalFA* represents a crucial parameter, as the SAR levels limit the lowest achievable TR. This is especially the case for the methods used in this work, which require values of *TotalFA* of the order of several tens to a few hundred degrees. In order to quantify the efficiency (ε) of each method to convert the employed RF-power into a phase accrual Ψ the following variable was defined and calculated:
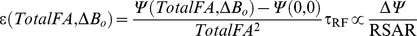
(1)where τ_RF_ is the total duration of the pulse.

### MR Hardware

Phantom studies and *in vivo* experiments of the human brain were performed at magnetic field strengths of 3.0 T and 7.0 T. For this purpose, methods A-E were implemented on a clinical 3.0 T MR-scanner (TIM Verio, Siemens Healthcare, Erlangen, Germany) and a whole body 7.0 T MR-scanner (Magnetom, Siemens Healthcare, Erlangen Germany), using a dedicated sequence development environment (IDEA, Siemens Healthcare, Erlangen, Germany). At 3.0 T a transmit/receive (TX/RX) birdcage coil (Siemens Healthcare, Erlangen, Germany) operating in the circular polarized (CP) mode was used (diameter = 27 cm, length = 31 cm). At 7.0 T a TX/RX birdcage coil (Siemens Healthcare, Erlangen, Germany) operating in the CP mode was used (diameter = 34 cm, length = 38 cm).

### Implementation of the *B_1_^+^* Mapping Techniques

The implementation comprises a standard 3D gradient-echo sequence, where the excitation is performed for each method by the non-selective RF-pulses sketched in Fig. 1 and described in the Theory section.

**Figure 4:**
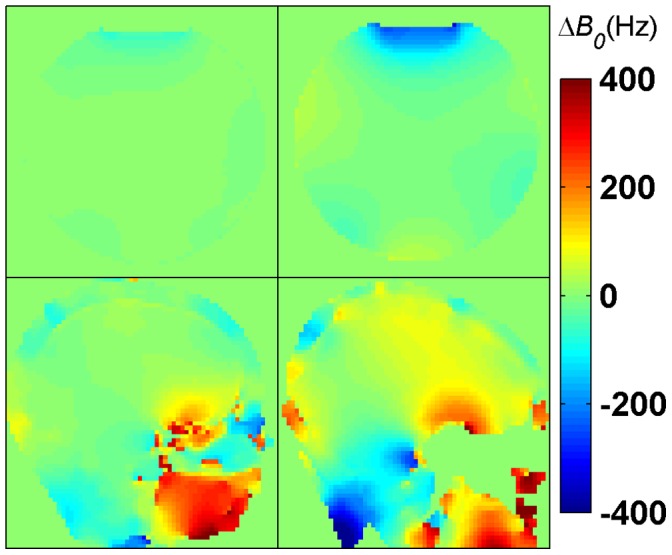
Δ*B_0_* maps in phantom and *in vivo* at 3.0 T and 7.0 T. Top: central axial partition of the 3D Δ*B_0_* maps obtained at 3.0 T (top-left) and at 7.0 T (top-right) in phantom with ΔTE = 2.5 ms; strong *B_0_* offsets are visible at the air-water interface in the upper part of the phantom. Bottom: central sagittal partition of the 3D Δ*B_0_* maps of the human brain obtained at 3.0 T (bottom-left) with ΔTE = 2.46 ms and at 7.0 T (bottom-right) with ΔTE = 3.06 ms; strong *B_0_* offsets are visible in the sphenoid sinuses area and, at 7.0 T, in the neck region. All maps are in hertz.

To reduce bulk motion effects the two images required by each method were acquired interleavedly. To examine and correct for variations in the main magnetic field (*B_0_*) across the object Δ*B_0_* maps were acquired. For this purpose a secondary gradient-echo readout was added to the sequence; the Δ*B_0_* maps (Fig. 4) were obtained from the subtraction of the two phase images acquired at different echo times (TE) [25].

**Figure 5:**
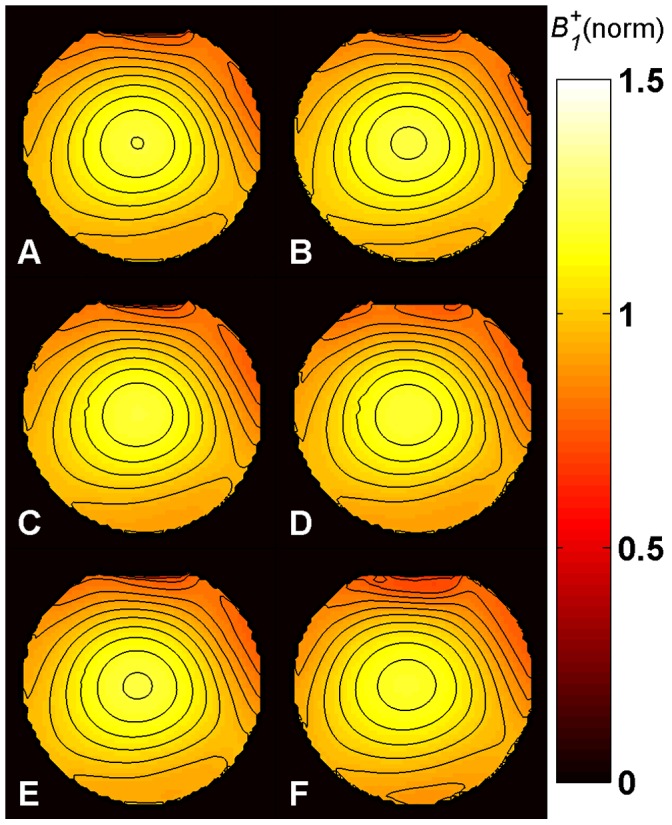
*B_1_^+^* maps in phantom at 3.0 T. Central axial partition of the 3D *B_1_^+^* maps obtained with methods **A**-**E** in phantom at 3.0 T using a birdcage TX/RX coil. Identical repetition times (TR = 30 ms) and SAR levels were used for all methods. The same partition of the *B_1_^+^* map acquired for comparison with the DAM with TR = 500 ms is also shown (**F**). All maps are normalized to their nominal *B_1_^+^* given in Table 1 in µT. The typical central spot of the birdcage TX/RX coil is visible. The contour plots (with contour increment of 0.05) show that the *B_1_^+^* distributions obtained from the phase-based methods are consistent with the DAM. Methods: **A –** *Optimized low-flip-angle method* **, B –** *Phase-sensitive method* ***,*** **C –** *ΦFA-CUP method* **, D –** *Bloch-Siegert method*, **E –** *Orthogonal-pulses method*, **F –** *Double Angle method*.

**Figure 6:**
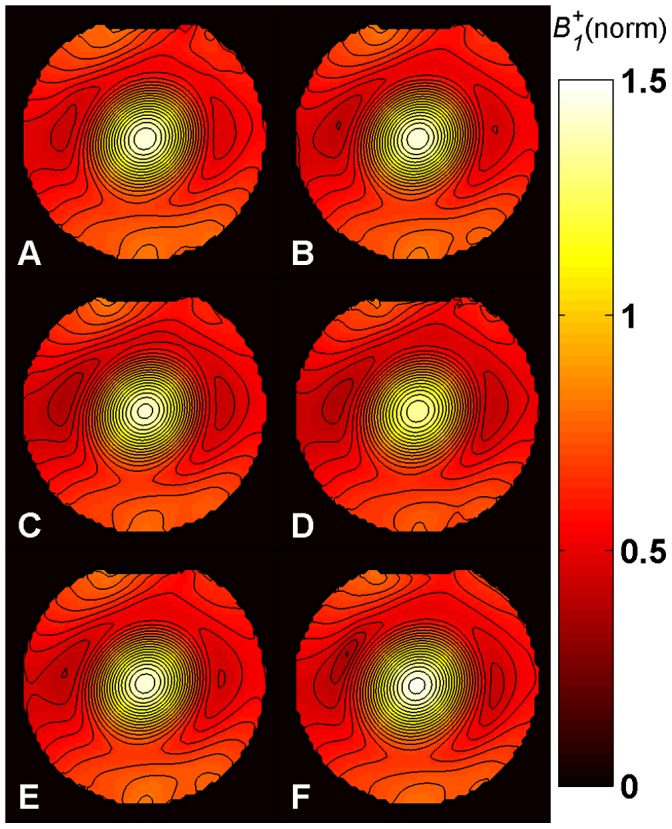
*B_1_^+^* maps in phantom at 7.0 T. Central axial partition of the 3D *B_1_^+^* maps obtained with methods **A**-**E** in phantom at 7.0 T using a birdcage TX/RX coil. Identical repetition times (TR = 110 ms) and SAR levels were used for all methods. The same partition of the *B_1_^+^* map acquired for comparison with the DAM with TR = 500 ms is also shown (**F**). All maps are normalized to their nominal *B_1_^+^* given in Table 1 in µT. The typical central spot of the birdcage TX/RX coil and the destructive interference patterns around it are visible. The contour plots (with contour increment of 0.05) show that the *B_1_^+^* distributions obtained from the phase-based methods are consistent with the DAM. Methods: **A –** *Optimized low-flip-angle method* **, B –** *Phase-sensitive method* ***,*** **C –** *ΦFA-CUP method* **, D –** *Bloch-Siegert method*, **E –** *Orthogonal-pulses method*, **F –** *Double Angle method*.

**Figure 7:**
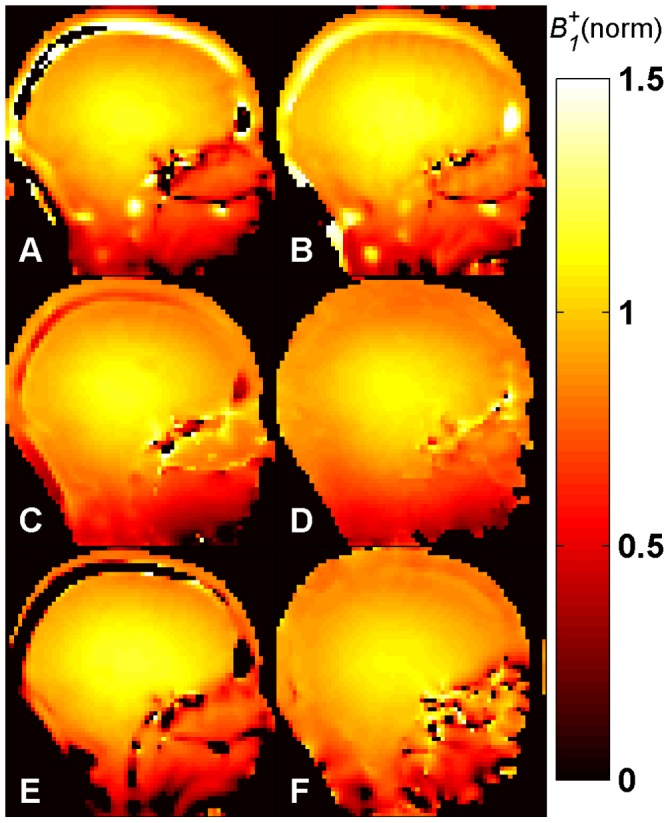
*B_1_^+^* maps *in vivo* at 3.0 T. Central sagittal partition of the 3D *B_1_^+^* maps of the human brain obtained with methods A-E *in vivo* at 3.0 T using a birdcage TX/RX coil. Identical repetition times (TR = 30 ms) and SAR levels were used for all methods. The central slice of the *B_1_^+^* map acquired for comparison with the 2D DAM, with TR = 6000 ms, is also shown (**F**). All maps are normalized to their nominal *B_1_^+^* given in Table 1 in µT. Methods: **A –** *Optimized low-flip-angle method* **, B –** *Phase-sensitive method* ***,*** **C –** *ΦFA-CUP method* **, D –** *Bloch-Siegert method*, **E –** *Orthogonal-pulses method*, **F –** *Double Angle method*.

**Figure 8:**
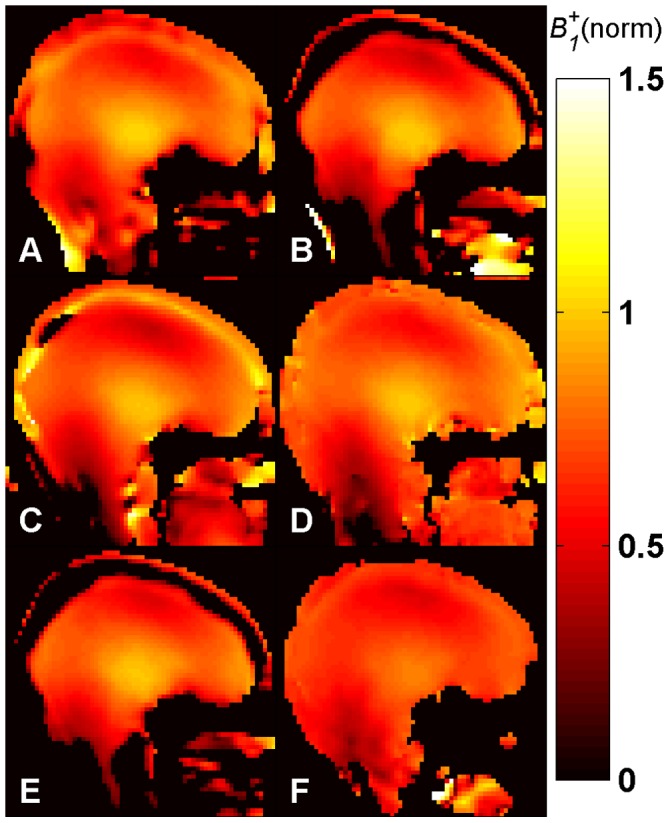
*B_1_^+^* maps *in vivo* at 7.0 T. Central sagittal partition of the 3D *B_1_^+^* maps of the human brain obtained with methods A-E *in vivo* at 7.0 T using a birdcage TX/RX coil. Identical repetition times (TR = 110 ms) and SAR levels were used for all methods. The central slice of the *B_1_^+^* map acquired for comparison with the 2D DAM, with TR = 6000 ms, is also shown (**F**). All maps are normalized to their nominal *B_1_^+^* given in Table 1 in µT. Methods: **A –** *Optimized low-flip-angle method* **, B –** *Phase-sensitive method* ***,*** **C –** *ΦFA-CUP method* **, D –** *Bloch-Siegert method*, **E –** *Orthogonal-pulses method*, **F –** *Double Angle method*.

*B_1_^+^* maps (Figs. 5–8) were calculated for each method from the measured phase accrual Ψ and the Δ*B_0_* map, using the corresponding curve of sensitivity of Fig. 3 as a lookup table and performing a linear 2D interpolation.

For comparison, a standard 3D gradient-echo technique was used to acquire three-dimensional *B_1_^+^* maps using the double-angle method (DAM) [4]. This required the acquisition of two magnitude images with nominal flip angles of α = 60° and 2α = 120° together with repetition times of TR>5*T_1_* [5].

### Specific Absorption Rate Adjustment

Each method uses a different RF-power level. Since the SAR represents the limiting factor for the minimum achievable TR at high field strengths for all methods, the RF-pulse amplitudes (i.e. the nominal *B_1_^+^*) were individually adjusted for each method in order to accomplish identical SAR levels, given a common repetition time. This corresponds to truncating the sensitivity curves of Fig. 3 to a *TotalFA* value which guarantees identical SAR levels for all methods. The TRs were adjusted in *in vivo* experiments - according to the volunteer weight - to achieve a nominal SAR level of 2.4 W/kg. This value corresponds to 75% of the SAR limit for the normal and first level operating modes for head imaging, as given by the IEC guidelines [26].

The nominal values of α, *TotalFA* and *B_1_^+^* for the five methods are reported in Table 1, together with the duration of the RF-pulses and the repetition times. The nominal *B_1_^+^* values are calculated starting from the reference voltage necessary to obtain a 1 ms rectangular π-pulse, and adjusted according to the duration τ and the *TotalFA* of the pulses of the five methods. The reported *B_1_^+^* intensity represents the average value within the pulse. Identical parameters were used for both phantom and *in vivo* experiments.

**Table 1:** Nominal values of the initial excitation angle (*p*0), flip angle of each sub-pulse (α), total flip angle (*TotalFA*), *B_1_*+ intensity, total duration of the RF-pulses, echo times (TE) and repetition times (TR) for the experiments performed in phantom and *in vivo*, at 3.0 T and 7.0 T, with methods A–E.

Method	*p0*	α	*TotalFA*	Total Duration	*B_1_^+^* intensity	TE	TR [ms] at 3.0 T	TR [ms] at 7.0 T
	[deg]	[deg]	[deg]	[µs]	[µT]	[ms]	phantom/*in vivo*	phantom/*in vivo*
**A**	-	20	120	600	13.0	1.55	30/30	110/110
**B**	-	40	120	600	13.0	1.42	30/30	110/110
**C**	20	15	140	490	14.6	1.47	30/30	110/110
**D**	20	250	270	4250	4.14	5.21	30/30	110/110
**E**	-	45	90	325	18.1	1.25	30/30	110/110
**DAM**	-	60	60	300	13.0	2.00	500/6000	500/6000
		120	120	600	13.0		500/6000	500/6000

Also the parameters for the comparison with the DAM are listed. The echo times are calculated relatively to the center of the first sub-pulse for each method.

### Phantom Studies

A synopsis of the imaging parameters used for phantom studies at 3.0 T and 7.0 T is shown in Table 1. The basic imaging parameters were kept constant for all methods, including: field of view FOV = (200×200×200) mm^3^, matrix size of 32×32×16 (plus zero-filling interpolation) and receiver bandwidth BW = 800 Hz/pixel. The echo times were set to the minimum possible value, which depends on the pulse duration of each method. For Δ*B_0_* mapping an inter-echo time of ΔTE = 2.5 ms was used. TRs needed to be prolonged at 7.0 T, in order to accomplish identical SAR levels as at 3.0 T.

A spherical phantom (18 cm diameter), filled with water and doped with 50 mM Na and 20 mM CuSO_4_, was prepared. This setup provides sufficient RF-loading and short *T_1_* relaxation time (at 3.0 T: *T_1_* ≈ 70 ms, *T_2_* ≈ 40 ms). The latter affords reasonable scan time for the DAM approach.

### Ethics Statement

For the *in vivo* feasibility study, two healthy subjects without any known history of neurovascular disease were included after due approval by the local ethical committee (registration number DE/CA73/5550/09, Landesamt für Arbeitsschutz, Gesundheitsschutz und technische Sicherheit, Berlin, Germany). Informed written consent was obtained from each volunteer prior to the study.

### *In vivo* Studies

Human brain imaging was performed at 3.0 T and 7.0 T in healthy subjects using the five phase-based methods (**A**-**E**) and the DAM. FOV was adjusted to (230×230×176) mm^3^ at 3.0 T and (210×210×160) mm^3^ at 7.0 T, in order to cover the whole brain with 16 sagittal partitions using a matrix size of 32×32×16 (plus zero-fill interpolation). The inter echo time for the Δ*B_0_* map was set to ΔTE = 2.46 ms at 3.0 T and ΔTE = 3.06 ms at 7.0 T, to make sure that fat and water are in phase for both TEs.

For the DAM approach only a central partition of the brain was acquired, since covering the whole brain would have required several hours of scan time: a constraint that is dictated by the *T_1_* of the brain (gray matter: *T_1_* ≈ 1800 ms, white matter *T_1_* ≈ 1000 ms at 3.0 T [27]), so that the repetition time was set to TR = 6000 ms.

## Results

### Numerical Simulations

The results derived from the simulations are shown in Figs. 2 and 3. The trajectories of **M** during excitation (Fig. 2) are used to qualitatively estimate the use of transverse magnetization. The curves displayed in Fig. 3 represent the sensitivities to *B_1_^+^* variations (expressed as the *TotalFA*) and frequency offsets (Δ*B_0_* in Hz). The frequency offset range is identical for all the curves, while the total flip angle ranges vary (*TotalFA* axis), as well as the phase accrual ranges (Ψ axis). Efficiency ε is used to combine the flip angle range and phase accrual characteristics in a single variable that supports a balanced comparison. The values of ε were calculated for each method at the center of the sensitivity curves using Eq. 1.

#### A – Optimized low-flip-angle method

This method revealed the lowest *B_1_^+^* sensitivity among all methods, with ε = 0.44 µs/deg. Its sensitivity curve presents a rather flat dependency upon frequency offsets. A discontinuity is observed for some combinations of Δ*B_0_* and *TotalFA* (Fig. 3A). In terms of usage of the transverse magnetization its composite pulse is equivalent to an excitation of 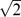
α (Fig. 2A).

#### B – Phase-sensitive method

This method shows the highest *B_1_^+^* sensitivity, with ε = 1.91 µs/deg. For frequency offsets exceeding a range of approximately ±500 Hz the phase accrual Ψ experiences a discontinuity (Fig. 3B). A folding, leading to non-unique phase information which could not be decoded into the *B_1_^+^* value, can also be observed outside of this range. This method employs the highest transverse magnetization, as the composite pulse is equivalent to a 90° excitation, when α = 90° (Fig. 2B).

#### C – ФFA-CUP method

This method has a high *B_1_^+^* sensitivity, with ε = 1.52 µs/deg. The dependency upon frequency offsets is more pronounced than in method **A**. This method does not present the discontinuities observed for methods **A** and **B** (Fig. 3C). The use of transverse magnetization is equal to *p0* (Fig. 2C). Its value can be chosen equal to the Ernst angle in order to optimize the signal, without affecting the *B_1_^+^* sensitivity. This is not possible for methods **A** and **B**.

#### D – Bloch-Siegert method

This method exhibits a low *B_1_^+^* sensitivity, with ε = 0.66 µs/deg, because it requires a much larger *TotalFA* compared to the other methods. This results also in a longer pulse duration, which manifests itself in a TE prolongation. The sensitivity curve of this method presents a rather modest dependency upon frequency offsets (Fig. 3D). The use of transverse magnetization depends only on the initial excitation *p0* (Fig. 2D), and can therefore be controlled, like for method **C**.

#### E – Orthogonal-pulses method

This method presents an intermediate *B_1_^+^* sensitivity, with ε = 0.82 µs/deg. Its sensitivity curve shows a non-negligible dependency upon frequency offsets. A reduced *B_1_^+^* sensitivity was observed for small flip angles versus the high flip angle regime (Fig. 3E). In terms of usage of the transverse magnetization the composite pulse used is equivalent to a flip angle larger than α (at small flip angles it is equal to 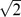
α, like for method **A**) (Fig. 2E).

### Phantom Studies

The results derived from phantom experiments at 3.0 T and 7.0 T are shown in Figs. 5 and 6. All maps present the typical behavior of a birdcage resonator, where *B_1_^+^* is higher at the center. At 7.0 T, due to destructive interference patterns, some areas around the center present a lower intensity.

The *B_1_^+^* maps obtained with methods **A**-**E** (Figs. 5–6, A–E) are compared to the DAM approach (Figs. 5–6, F).

*B_0_* inhomogeneities can be observed at the air-water interface from the Δ*B_0_* maps shown in Fig. 4 (top), especially at 7.0 T. However, the fit performed using the sensitivity curves provides a very good estimation of *B_1_^+^* in these regions. Even for the *phase-sensitive method* (**B**), the *ФFA-CUP method* (**C**) and the *orthogonal-pulses method* (**E**) which are most sensitive to frequency offsets. This is confirmed by the contour plots in Figs. 5 and 6. Compared to the DAM no *B_1_^+^* distortion can be observed in these areas.

All methods revealed sufficient signal, as the *T_1_* of the phantom was short enough (*T_1_* ≈ 70 ms) to allow recovery of the magnetization.

### *In vivo* Studies

The results of the human brain studies at 3.0 T and 7.0 T are summarized in Figs. 7 and 8. The results obtained with methods **A**–**E** (Figs. 7–8, A–E) for brain regions are in agreement with the DAM (Figs. 7–8, F). The typical *B_1_^+^* peak of a birdcage coil can be observed at the center of the brain both at 3.0 T and 7.0 T. At 7.0 T a region of signal void due to destructive interference is visible in the area of the cerebellum. It should be noted that all methods except the *Bloch-Siegert method* (**D**) present some regions where the *B_1_^+^* estimation is not correct, both at 3.0 T and at 7.0 T. This is due to the 3.5 ppm chemical shift between fat and water (corresponding to a resonance frequency difference of 150 Hz/T). The individual phases of water and fat signals are affected by the presence of *B_1_^+^* inhomogeneities and frequency offsets, as demonstrated by the sensitivity curves (Fig. 3). Since the signal from each pixel is given by the complex sum of these two components, the resulting phase is decoded into a wrong *B_1_^+^* value during the fitting. In fact, the Δ*B_0_* maps shown in Fig. 4 (bottom) do not account for this effect, as they were acquired with fat and water in phase. Due to air-tissue interfaces, strong *B_0_* offsets were observed in the sphenoid sinuses area, extending into the interior of the brain. The correction fit performs correctly in this region.

At 7.0 T the *Bloch-Siegert method* (**D**) shows SNR loss in the regions with short T_2_^*^, such as the areas nearby the bones.

## Discussion

In this work five phase-based methods used for *B_1_^+^* mapping have been examined carefully at magnetic field strengths of 3.0 T and 7.0 T. The characteristics of each method were analyzed by means of numerical simulations, phantom studies and *in vivo* experiments.

Although all methods have in common the use of a *complex* RF-pulse (composite or off-resonance pulse) for excitation, in conjunction with the same gradient-echo readout scheme, it is shown here that the five methods exhibit different sensitivities to *B_1_* ^+^ inhomogeneities and frequency offsets. Furthermore they make different use of transverse magnetization and hence reveal different SNR, depending on the TR/*T_1_* ratio. For these reasons, the quality of the *B_1_^+^* maps obtained from each method depends on the specific experimental conditions (*T_1_*, T_2_^*^, frequency offset and dynamic range of *B_1_^+^* in the region of interest) and on the parameters settings (TR, TE, flip angles and duration of the RF-pulse).

In this work, a fixed TR and an identical SAR level were used for all methods to compare their performances under fast imaging conditions. This approach was chosen deliberately since SAR limits dictate the minimum TR achievable, especially at high and ultrahigh magnetic field strengths. Within these limits all phase-based methods support short TR, since full relaxation of the longitudinal magnetization is not required prior to each excitation. Due to this SAR restriction the feasibility of using short TRs varies for each method, and depends primarily on the characteristics for the small nominal flip angle regime. The main challenge in the small flip angle regime is achieving enough *B_1_^+^* sensitivity, given by the phase accrual. In order to quantify the ability of each method to convert the employed RF-power into the *B_1_^+^* information, efficiency, which is defined as the specific phase accrual per unit SAR, was examined carefully. The *phase-sensitive method* (**B**) showed the best efficiency, followed by the *ФFA-CUP method* (**C**), the *orthogonal-pulses method* (**E**), the *Bloch-Siegert method* (**D**) and the *optimized low-flip-angle method* (**A**). According to our results, the use of very short TRs in conjunction with methods which have a low efficiency results in highly noise-corrupted *B_1_^+^* maps [28].

Another important characteristic for the quality of the resulting *B_1_^+^* maps is represented by the consumption of longitudinal magnetization. Since the SNR of phase images is directly proportional to that of magnitude images, the amount of longitudinal magnetization available at each repetition should support the phase measurements with enough signal, in order to provide reliable phase images. In this regard, the *ФFA-CUP method* (**C**) and the *Bloch-Siegert method* (**D**) are superior to the others. In fact the pulses used by these two methods include an initial excitation which is independent from the *B_1_^+^* sensitization. To optimize the SNR this initial excitation can be set to the Ernst-angle. According to the simulations the *phase-sensitive method* (**B**), which provides the highest efficiency, uses the largest transversal magnetization and may be affected by a severe SNR drop when the TR/*T_1_* ratio is too small [28]. The large use of transverse magnetization could be useful if another reading module, such as EPI, is used. However this approach bears the risk to result in geometric distortion artifacts, due to magnetic field inhomogeneities, which pose a significant challenge [29], especially at 7.0 T.

It should be noted that *B*_1_^+^ mapping is not a problem limited to proton MRI. For instance the *phase-sensitive method* (**B**) has been applied to ^23^Na MRI [30], where the low MRI signal and the short relaxation time *T_1_* require a large use of transverse magnetization, without incurring in saturation effects. On the other hand, the *optimized low-flip-angle method* (**A**) and the *ФFA-CUP method* (**C**) were originally proposed for low field MRI using hyperpolarized ^3^He [14], [17], [18], were the frequency offsets are not significant, and the longitudinal magnetization needs to be preserved.

For all methods, except the *Bloch-Siegert method* (**D**), the knowledge of Δ*B_0_* is required to perform a correct fitting to obtain the *B_1_^+^* magnitude. This may require additional scan time. However, the acquisition of a second echo is feasible and affords the Δ*B*_0_ mapping with no extra scan time. In any case, localized *B_0_* shimming would further improve the results. For these methods the presence of the fat-water chemical shift may affect the estimation of *B_1_^+^*, as the Δ*B*_0_ maps do not account for this effect. In this case a fat-water separation approach could be eventually applied to remove artifacts.

All the five methods revealed that the sensitivity change induced by frequency offsets (Δ*B_0_* direction) would be reduced for shorter pulse durations. Also the frequency offsets, at which the discontinuities observed for the *optimized low-flip-angle method* (**A**) and the *phase-sensitive method* (**B**) occur, could be shifted away from the chosen range of ±1 kHz, as they are inversely proportional to the pulse duration. On the downside this approach would hamper the efficiency ε (Eq. 1), since the SAR is increased for shorter RF-pluses due to the increase in the peak power necessary to achieve the same flip angle.

Among the methods used here, the *Bloch-Siegert method* (**D**) was found to be the least sensitive to Δ*B_0_* offsets and chemical shift effects, due to its flat sensitivity curve in the frequency offset direction.

Unlike all the other methods, the *Bloch-Siegert method* (**D**) supports also 2D mapping. This can be beneficial when time constraints do not allow for a full 3D acquisition; for example for *B_1_^+^* mapping of the heart, where scan time constraints dictated by cardiac and respiratory motion need to be managed carefully. On the other hand the *Bloch-Siegert method* (**D**) presents a smaller efficiency than the *phase-sensitive method* (**B**), the *ФFA-CUP method* (**C**) and the *orthogonal-pulses method* (**E**), and requires the longest pulse duration among all the methods. This feature results in SNR degradation for short T_2_^*^ regions, such as interfaces with strong susceptibility gradients.

As our work is focused on the excitation pulse, results are derived using a standard 3D gradient-echo sequence, a Cartesian *k*-space sampling scheme and a single channel TX/RX coil. However, all the methods are inherently compatible with other 3D-imaging modules and *k*-space sampling schemes, as long as the phase information is preserved. Therefore all the methods can be accelerated using multi-echo techniques, or *k*-space undersampling techniques. This can be useful for *B_1_^+^* mapping applications in other organs, including cardiac or abdominal MRI where physiological motion constraints dictate the viable window of data acquisition.

### Conclusion

The *B_1_^+^* mapping techniques examined here provided characteristics which underline the capabilities of phase-based methods, including the scan time advantage over conventional magnitude-based *B_1_^+^* mapping methods. All presented methods can be adjusted to provide enough *B_1_^+^* sensitivity without exceeding the clinical SAR limits. However, some characteristics, such as the sensitivity to *B_1_^+^* inhomogeneities and frequency offsets and the consumption of longitudinal magnetization, are different for each method. This has an impact on the performance, depending on the specific experimental conditions.
